# A Comprehensive Optimization Course of Antimony Tin Oxide Nanofiller Loading in Polyamide 12: Printability, Quality Assessment, and Engineering Response in Additive Manufacturing

**DOI:** 10.3390/nano14151285

**Published:** 2024-07-30

**Authors:** Nektarios K. Nasikas, Markos Petousis, Vassilis Papadakis, Apostolos Argyros, John Valsamos, Katerina Gkagkanatsiou, Dimitrios Sagris, Constantine David, Nikolaos Michailidis, Emmanuel Maravelakis, Nectarios Vidakis

**Affiliations:** 1 Division of Mathematics and Engineering Sciences, Department of Military Sciences, Hellenic Army Academy, Vari, 16673 Athens, Greece; nasikas@sse.gr; 2 Department of Mechanical Engineering, Hellenic Mediterranean University, 71410 Heraklion, Greece; markospetousis@hmu.gr (M.P.); valsamos@hmu.gr (J.V.); tm6759@edu.hmu.gr (K.G.); 3 Department of Industrial Design and Production Engineering, University of West Attica, 12243 Athens, Greece; v.papadakis@uniwa.gr; 4 Institute of Electronic Structure and Laser, Foundation for Research and Technology-Hellas, N. Plastira 100m, 70013 Heraklion, Greece; 5 Physical Metallurgy Laboratory, Mechanical Engineering Department, School of Engineering, Aristotle University of Thessaloniki, 54124 Thessaloniki, Greece; aargyros@auth.gr (A.A.); nmichail@auth.gr (N.M.); 6 Centre for Research & Development of Advanced Materials (CERDAM), Center for Interdisciplinary Research and Innovation, Balkan Centre, Building B’, 10th km Thessaloniki-Thermi Road, 57001 Thessaloniki, Greece; 7 Department of Mechanical Engineering, International Hellenic University, Serres Campus, 62124 Serres, Greece; dsagris@ihu.gr (D.S.); david@ihu.gr (C.D.); 8 Department of Electronic Engineering, Hellenic Mediterranean University, 73133 Chania, Greece; marvel@hmu.gr

**Keywords:** polyamide 12 (PA12), antimony tin oxide (ATO), material extrusion three-dimensional printing (MEX 3D-P), mechanical performance

## Abstract

This study aimed to investigate the potential of antimony-doped tin oxide (ATO) as a reinforcing agent for polyamide 12 (PA12) in 3D printing by examining four mixtures with varying ATO concentrations (2.0 to 8.0 wt.%, with a 2.0 wt.% interval). These mixtures were used to fabricate filaments for the manufacturing of specimens through the material extrusion method. The mechanical properties of the resulting PA12/ATO composites and PA12 pure samples were evaluated through tensile, Charpy impact, flexural, and microhardness tests. Additionally, rheology, structure, morphology, thermal properties, pore size, and consistency in the dimensions of the samples were evaluated. Thermogravimetric analysis, along with differential scanning calorimetry, scanning electron microscopy, energy-dispersive and Raman spectroscopy, and micro-computed tomography, were conducted. The results were correlated and interpreted. The greatest reinforcement was achieved with the PA12/ATO 4.0 wt.% mixture, which exhibited a 19.3% increase in tensile strength and an 18.6% increase in flexural strength compared with pure PA12 (the control samples). The Charpy impact strength and microhardness were also improved by more than 10%. These findings indicate the merit of composites with ATO in additive manufacturing, particularly in the production of components with improved mechanical performance.

## 1. Introduction

Additive manufacturing (AM) refers to a variety of methods suitable for the production of objects that are connected by the operating principle of creating a 3D-printed object by depositing layer after layer of the selected material. AM can deliver complex shapes and microstructures with a high degree of automation, accuracy, and reproducibility [1]. Some well-known AM techniques include 3D printing [2,3,4,5,6], Selective Laser Sintering (SLS) [7], Fused Filament (Deposition) Fabrication (Modeling) (FFF/FDM) [8], Laser Metal Deposition (LMD) [9], stereolithography (SLA) [10], and Laminated Objective Manufacturing (LOM) [11]. The technology of 3D printing produces objects using the opposite principle to that of subtractive manufacturing (e.g., drilling, milling, broaching, sawing) [12,13]. Possible applications of AM can be applied to objects in aerospace [14,15,16,17,18,19,20,21,22], automotive [15,16,23,24], medical [25,26,27,28], food [29], and energy fields [30,31,32,33].

Some of the most well-known AM materials include Acrylonitrile Butadiene Styrene (ABS) [34], Polylactic Acid (PLA) [35], Polyethylene Terephthalate Glycol (PET-G) [36], and polyamide 12 (PA12) [37]. PA12 is a polyamide known for its strength, impact strength, toughness, and fracture resistance [38]. Due to its characteristics, it is a popular polymer in thin films, membranes, and packaging applications [39,40]. Polyamides have been utilized in composites, along with different types of fillers, for purposes such as electrospinning [41,42,43] and dental applications [44,45,46]. In 3D printing, its performance in mechanical testing has been reported [47,48] and optimized [49]. Its applications in 3D printing include membranes [50] and the development of multifunctional composites for medical implants, among other medical applications [51]. For the development of the composites for medical applications in MEX 3D printing, additives that have been evaluated include carbon black [52], cuprous oxide [53], titanium nitride (a ceramic filler) [54], and silver [55,56]. Cuprous oxide and silver additives also induced biocidal properties in the composites. Other additives for various applications, such as alumina [38] and carbon fibers [57,58,59], have been evaluated as well, focusing on the improvement of the resilience of the 3D-printed items.

Some of the characteristics of antimony tin oxide (ATO) are its low resistivity, high surface composition, and high optical transmission. This may differ depending on the technique used to prepare the nanoparticles [60,61,62]. It is used in thin films [63], optical, and window applications [64,65], due to its properties. Therefore, it is utilized in solar cells, panel displays, and electromagnetic interference shielding [66,67,68,69]. In 3D printing, it has been evaluated as a reinforcement additive in polymeric matrices such as Polypropylene (PP) [70], Polyethylene terephthalate glycol PETG [71], and Acrylonitrile Butadiene Styrene ABS [62]. Overall, its use with this manufacturing technology is still limited, based on the search carried out in the existing bibliography.

The current study focused on investigating the impact of ATO filler on the behavior of PA12. Based on the bibliography review, no similar research exists with PA12/ATO composites in material extrusion (MEX) 3D printing. The 3D printing of polyamides is challenging by itself [51,72]. Polyamides are used in specific types of applications, and increasing their strength can lead to the production of more robust parts with reduced weight and material use. The additives are also suitable for different types of applications, and their effects differ in each polymeric matrix, as the literature reports. Therefore, there is a necessity to study the polymer/filler combinations individually, especially since additives can harm the qualities of the matrix, such as its thermal stability or rheology, among others. In this research, first, the materials (in their raw form) were prepared in the right quantities and then mixed immediately before they were processed further, aiming to create the corresponding materials used for 3D printing. These produced filaments were then used to examine their mechanical properties and fabricate three-dimensional (3D) printed specimens. The samples from the manufactured specimens underwent multiple tests to investigate their tensile, Charpy impact, flexural, and microhardness (M–H) characteristics. The rheological and thermal behaviors of the PA12/ATO nanocomposites and PA12 pure were put under a microscope. TGA and DSC were part of the thermal investigation, whereas viscosity and material flow rate (MFR) were conducted for rheometric evaluation. Scanning electron microscopy (SEM) was employed, and pictures of the fracture and side structure of the specimen were acquired. Furthermore, the fabricated specimens underwent μ-computed tomography (μ-CT) to determine the geometrical deviation and voids in relation to the respective designed models. All nanocomposites exhibited improved behavior, especially PA12/ATO 4.0 wt.%. The baseline of the present work is as follows:

1. The effect of ATO as a filler on the behavior of the PA12 matrix material was investigated.
2. The creation of PA12/ATO composites suitable for utilization in the MEX 3D printing (3D-P) process with the aim of producing objects with reinforced mechanical characteristics.
3. Analyzing the influence of ATO on critical aspects of the fabricated parts, such as thermal stability, morphology, and quality metrics, such as the spatial consistency and the pore size and characteristics of the 3D printing specimens.

## 2. Materials and Methods

**Figure 1:**
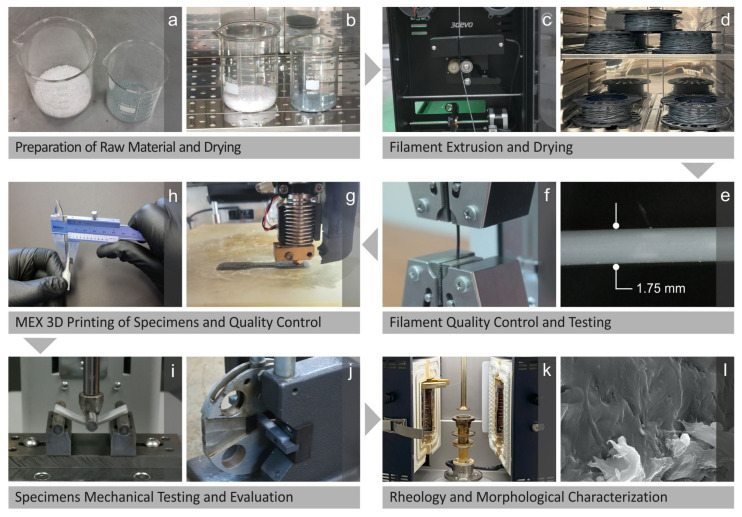
(**a**) The materials in their raw form being prepared and then (**b**) dried, (**c**) filaments being extruded, as well as (**d**) dried, (**e**) quality inspected and (**f**) mechanically tested, (**g**) specimens being MEX 3D printed, (**h**) quality controlled, (**i**,**j**) mechanically tested and evaluated, (**k**) rheological examination, (**l**) structural characterization of the samples.

The materials (in their raw form) were initially prepared in the right quantities and placed in a laboratory oven to dehydrate (Figure 1a,b) before being mixed. Separate mixtures were prepared for each composite with different filler content. The mixtures were supplied individually in a filament extruder, and the derived products were left in an oven for dehydration (Figure 1c,d). The filaments were then inspected for quality and tested mechanically (Figure 1e,f). The investigation proceeded by using filaments to create 3D-printed specimens through material extrusion and then inspecting their quality (Figure 1g,h), as well as testing and evaluating their mechanical behavior (Figure 1i,j). In addition, the rheology of the samples was examined, and morphological characterization was conducted (Figure 1k,l).

### 2.1. Materials

Fine grains of polyamide 12 (PA12) were supplied by AESNO/TL class Arkema S.A. (Rilsamid/PA12/AESNOTL, Colombe, France). According to the material’s datasheet, the Melt volume flow rate of the PA12 used is 8 cm^3^/10 min (ISO 1133). Sigma Aldrich (St. Louis, MO, USA) sourced the spherical nanoparticles of ATO (surface area 47 m^2^/g, particle size < 50 nm, purity ≥ 99.5%, Sb-doped SnO_2_, tin(IV) oxide, 89–93%), antimony pentoxide, 7–11%.

### 2.2. Mixture Preparation, Filament Extrusion, and Specimen 3D-P

First, the quantities of the raw materials were measured according to the desired concentrations required for each mixture. Different mixtures of the raw materials were prepared, one for each composition (filler content). The raw materials were weighed with an electronic high-precision scale at the required amounts. They were put in individual containers, one for each composition. Four different mixtures were composed of PA12/ATO (2.0 wt.%–8.0 wt.%) with a 2.0 step (no other additives were used in the mixtures). All the samples were placed in an oven to dehydrate at approximately 80 °C overnight. The raw materials were mixed for 30 min to produce composite powders with as good a distribution of ATO particles as possible in the PA12 grains. These mixtures were then fed to the extruder to produce the respective filament of each composition.

Filament extrusion was performed by utilizing a special extruder (3D Evo Composer, Utrecht, The Netherlands). A detector was also used to measure the diameter of the filament during the production procedure and implement micro-adjustments to the extrusion speed, when necessary, with the aim of achieving a steady diameter. The total number of filaments was extruded, having a diameter value in a range adequate for MEX 3D printing (1.65 and 1.85 mm). To determine a proper filler percentage range, the filler’s percentage was increased step-by-step. The samples of each filler content were tested for their mechanical response. When the tensile and flexural properties began to decrease, the filler percentage no longer increased. As will be shown below, the tensile and flexural properties were constantly decreased by increasing the ATO content in the prepared composites beyond 4.0 wt.%. This was not the case for the impact tests. The reason for this is explained in the discussion section. This indicated nanoparticles’ saturation in the matrix, so no further improvement in the mechanical properties was expected with higher loadings [73,74]. The exact threshold determination fell far away from the core scope of this work.

The literature was used to determine the optimum parameters for extrusion [54]. The filaments were inspected for their quality and left to dry in a preheated oven at 80 °C overnight before being fed to the 3D printers. The PA12/ATO samples were produced using an FFF apparatus. More specifically, the Funmat/HT 3D printer from Intamsys Tech. Co. Limited, located in Shanghai, China, was employed.

### 2.3. Morphological and Chemical Investigation of the ATO Material along with the PA12/ATO Composites

The morphology of vertical and fracture regions of the examples was investigated by SEM employing a field-emission apparatus model named JSM-IT700HR by the JEOL company, located in Tokyo, Japan. Moreover, the compositional characteristics were inspected by Energy Dispersive Spectroscopy (EDS) coupled with the SEM instrument. For the aforementioned analyses, the samples were gold-coated to prevent charging issues, and the apparatus was set at high-vacuum mode, with the acceleration voltage set at 5 kV.

**Figure 2:**
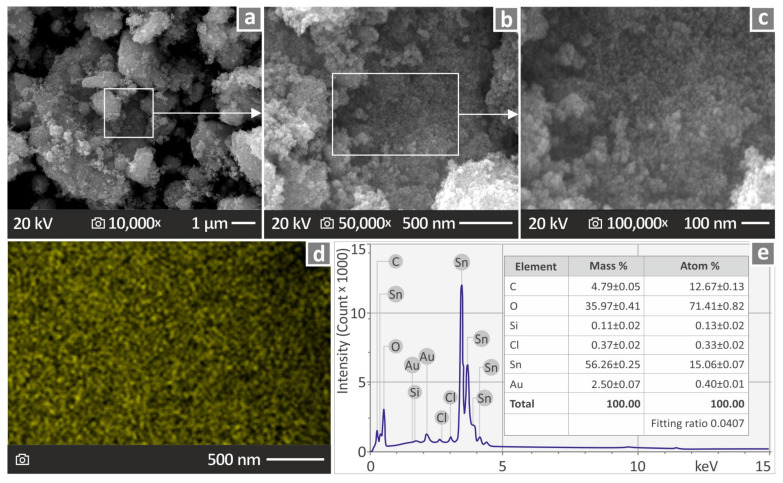
ATO-related (**a**–**c**) SEM illustrations at several magnifications, namely 10,000×, 50,000×, and 100,000× correspondingly, (**d**) EDS MAP picture (Sn element), and (**e**) chemical composition shown through EDS analysis.

Figure 2 presents the images acquired from SEM and EDS analyses of the ATO nanopowder. Figure 2a–c present SEM images magnified ×10,000, ×50,000, and ×100,000, respectively. Figure 2d shows an EDS mapping image (Sn element), and Figure 2e presents the EDS analysis results based on the levels of its chemical elements. It can be observed that Sn was the element found in the greatest amount, which is in agreement with the composition of the nanoparticles, according to the manufacturer.

### 2.4. Micro-Computed Tomography (μ-CT)

μ-CT technology was employed to assess the dimensional deviation and voids of the produced samples to evaluate the effect of the additive manufacturing process on the structural behavior of the samples. An HV Tomoscope Compact/225  kV Micro-Focus CT digital scanner (Werth GmbH, Giessen, Germany) was used. A 1024 over 1024 pixel sensor, accompanied by software named VG Studio/MAX/2.2 (Volume Graphics, Heidelberg, Germany), was engaged to post-process the derived data.

For dimensional actual/nominal geometries, a 75 L settings setup characterized by 72.58 μm X-axis resolution and 72.65 μm Y-axis resolution was utilized. For the porosity, a 16 L settings setup characterized by 15.46 μm X-axis resolution and 15.49 μm Y-axis resolution was used, while 1600 sections/revolution were acquired for all instances.

### 2.5. Mechanical Properties Testing

Several experiments were performed in an effort to elucidate the mechanical performance of the samples, including those related to the uniaxial tension, flexural, impact resistance after Charpy, and microhardness (M–H) properties. The respective standards that were considered are: ASTM D638-02a [75] (V-shaped tensile sample with a thickness of 3.2 mm), ASTM/D790-10 [76] (three-point bending with a support span of 52.0 mm), ASTM D6110-04 [77], and ASTM E384-17 [78].

For the tensile tests, the Imada MX2 type (Imada Incorporation, Northbrook, IL, USA), equipped with standardized grips, was employed. The same device was exploited for the bending tests; instead, the flexural setup was placed according to the right standard. A 10 mm/min speed of testing was applied in both the tensile and the flexural tests, in accordance with the respective standards. The Imada MX2 machine was equipped with a 2.5 KN load cell. The force was gradually increased until the samples failed in the tensile test and until 5% strain was developed in the flexural tests, following the instructions of the respective standard. In the flexural test, the ASTM D790 instructs that if the sample has not failed at 5% strain, the test can be terminated, as it is considered completed.

For impact testing, a Terco MT 220 Charpy impact device (Terco, Stockholm, Sweden) was employed. The pendulum’s mass was 2 kg, and its length was 0.39 m. For the microhardness test, an Innova Test 300 Vickers apparatus (Innovatest, Europe BV, Maastricht, The Netherlands) was used. The surfaces of the specimens were fully polished with an applied force of 100 gF, and the indentation duration was set to 10 s. All tests were performed in room conditions for temperature (22 °C) and humidity (~55%).

**Figure 3:**
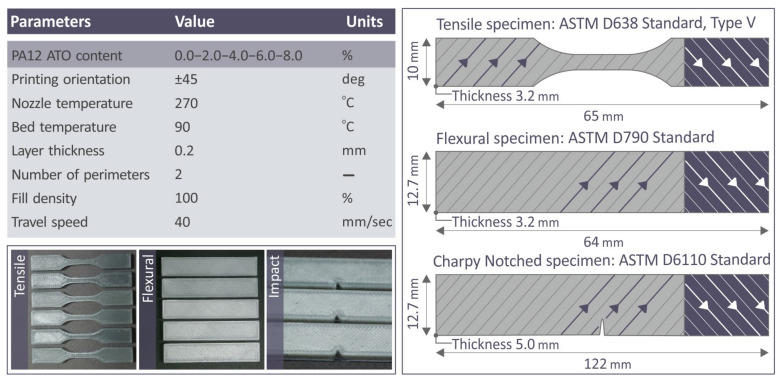
The 3D printing parameters and their values are listed on a board; samples of the 3D-P specimens fabricated for flexural, tensile, and impact specimens are presented as well; and the models of the same specimens are accompanied by their dimensions and follow ASTM standards. In the illustrations, the arrows shown in the samples present the shape selected for the infill pattern, in which the lines of the strands swift from +45 degrees angle to −45 degrees angle in the various layers, aiming to reduce the anisotropy within the composites.

Figure 3 shows the chosen 3D printing details regarding various composites synthesized, along with their values. The samples from the printed specimens used for the tensile, flexural, and impact tests are also shown in Figure 3. Their dimensions and ASTM standards were also presented.

### 2.6. Raman Measurements

Raman Spectroscopy measurements were conducted using a confocal Raman Spectrometer (LabRAM HR; HORIBA Scientific, Kyoto, Japan) under controlled laboratory conditions. The setup employed a 532 nm primary laser line with a 90 mW power output, moderated by a 5% Neutral Density filter to reduce the laser power incident on the sample. For excitation and imaging, a 50× objective lens (LMPlanFL N, Olympus, Tokyo, Japan) with a numerical aperture (NA) of 0.5 and a working distance of 10.6 mm was utilized.

The spectral window ranged from 40 to 3900 cm^−1^, and the spectrometer grating had 600 grooves/mm, providing a spectral resolution of 2 cm^−1^. Each measurement point had an exposure time of 10 s, with data acquisition consisting of five accumulations per point to ensure statistical reliability. The imaging resolution was 1.7 μm lateral and 2 μm axial. Laser power on the sample surface was measured at 2 mW, and post-analysis visual inspection through the microscope confirmed no discoloration or degradation due to laser irradiation.

The raw Raman data were processed using LabSpec software v.6 (HORIBA, Kyoto, Japan) with the following steps: removal of cosmic rays, signal noise reduction using a 5-point kernel, data cropping in the range of 750–3150 cm^−1^, background removal using an 8th-degree polynomial, spectral recalibration using the 1292 cm⁻^1^ peak, and normalization by the maximum peak intensity. To highlight differences, the pure PA12 spectrum was subtracted from all PA12/ATO samples.

### 2.7. Rheometric Investigation

To conduct the rheometric tests, ASTM D1238-13 [79] for melt flow rate (MFR) was considered. A DHR-20 Discovery Hybrid Rotational Rheometer (TA Instruments, New Castle, DE, USA) was employed, which was equipped with an Environmental Test Chamber featuring a parallel-plate setup of 25 mm diameter (~1 mm distance), responsible for properly regulating the temperature. The test temperature was 240 °C, and excessive heating or decomposition was prevented by setting the acquisition time to 20 s for each measurement point. The chosen temperatures and presently determined pressures were assessed by MFR and rotational rheometric testing. MFR measurements were conducted in accordance with international standards (ASTM D1238-13) [79]. The specific conditions used were a mass of 2.16 kg and a temperature of 235 °C. We did not perform oscillatory rheology measurements. Instead, we conducted flow sweep rheology experiments within a shear rate range of 0.002 to 50 s^−1^. This range was determined based on preliminary flow-run experiments.

### 2.8. Thermal Behavior

The thermal performances of the pure PA12 and PA12/ATO composites’ samples were determined by TGA and DSC. The apparatus used for TGA analysis was a Diamond Perkin Elmer (Waltham, MA, USA). The temperature ranged between 40 and 550 °C, and the temperature increase rate was 10 °C/min. The mass of the sample was approximately 8 mg (of filament), and the gas flow was adjusted to 200 mL/min. DSC was conducted using a Discovery Series DSC-25 DSC calorimeter (TA Instruments, New Castle, DE, USA) coupled with an RSC-90 Refrigerated Cooling System. The mass of the sample was approximately 6 mg (of filament), and the gas flow was adjusted to 50 mL/min. An inert environment prevailed during the TGA and DSC experiments, along with inert N_2_ (nitrogen high-purity gas).

## 3. Results

### 3.1. Raman Spectra

**Figure 4:**
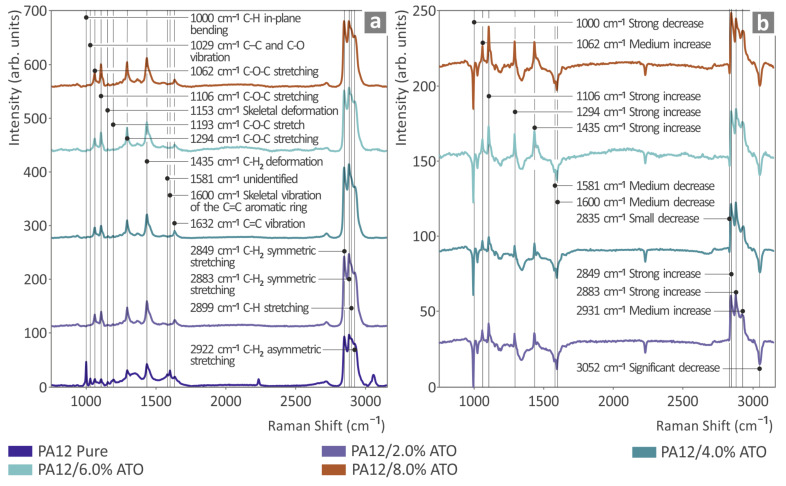
(**a**) Raman analysis information regarding PA12 pure, PA12/ATO 2.0 wt.%–8.0 wt.% ATO, and (**b**) the derived subtraction results between PA12 pure and the total amount of PA12/ATO composites.

Figure 4a depicts the Raman spectral profiles of the unfilled PA12 and all PA12/ATO mixtures, while Figure 4b shows the outcomes derived after deducting the unfilled PA12 from the PA12/ATO Reman spectral profiles. In the Supplementary Materials, a table lists the (Raman) peaks of the unfilled PA12 sample, as derived through the existing bibliography [80,81,82,83,84,85,86,87]. By introducing ATO to PA12, the Raman lines at 1000, 1029, 1581, and 1600 cm^−1^ of pure PA12 were lost. Moreover, the broad photoluminescence across the spectral sensitivity range increased with increasing concentrations of ATO.

ATO as a filler for PA12 provided some Raman spectral changes when subtracted from the pure PA12 spectrum. Particularly, Raman intensity decreased at 1000, 1229, 1581, and 1600 (until they disappeared from the PA12 pure spectrum) and at 2835 and 3052 cm^−1^. In contrast, Raman signal intensity increase was detected at 1062, 1106, 1294, 1435, 2849, and 2883 cm^−1^. It is important to note that three Raman lines were observed (new lines), which were not clearly distinguishable at the PA12 pure Raman spectrum. Particularly, the Raman line at 2835 cm^−1^ showed a gradual intensity drop, the Raman line at 3052 cm^−1^ showed a significant intensity decrease, and the Raman line at 2931 cm^−1^ presented an intensity increase. The total amount of spectral change is depicted in Table 1.

ATO addition should present some changes in the low Raman spectral range, below 1000 cm^−1^, but in our samples and with our instrumentation, we were unable to observe any changes in the specific Raman lines (621 and 757 cm^−1^) [88]. On the other hand, the observed changes in Table 1 could possibly be explained by the creation of new bonds between Sb, SnO, and PA12.

### 3.2. Thermal Investigation Results

**Figure 5:**
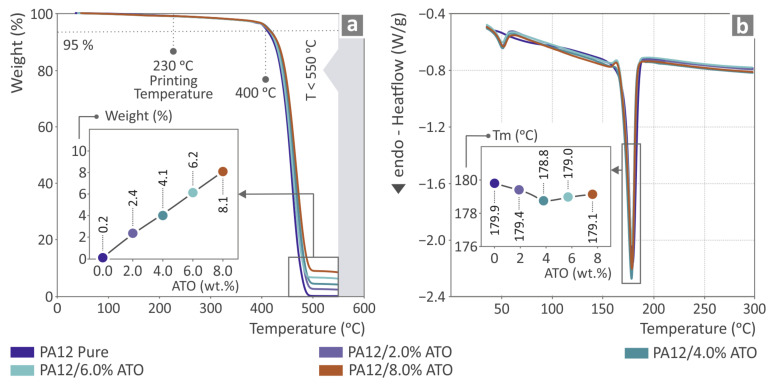
Thermal properties evaluation regarding PA12 pure and PA12/ATO 2.0–8.0 wt.% composites, through the possession of (**a**) TGA graphs and (**b**) DSC graphs, both of them accompanied by graphs showing the percentage of weight versus the ATO quantity and the Tm values as to the ATO quantity, respectively.

Figure 5 shows the thermal investigation results, namely the TGA graph shown in Figure 5a and the DSC graph shown in Figure 5b, of all the PA12/ATO nanocomposites and PA12 pure, accompanied by inset graphs of the residual weight and Tm as to the ATO quantity, respectively. The TGA graph shows that all samples exhibit almost the same behavior, while the inset graph indicates that the residual weight also increased and agrees with the ATO content in the nanocomposites. Table 2 presents the Initial Decomposition Temperature (IDT) at 10% and 50% mass loss, as it was derived from the TGA measurements.

The inset in the DSC graph indicates that the lowest Tm value was detected for PA12/ATO 4.0 wt.%. In the measurements, we have not calculated the degree of crystallinity because a clear exothermic peak for the crystallization temperature (Tc) was not observed in the DSC measurements. As a result, it was not possible to accurately determine these parameters.

### 3.3. Rheometric Results

**Figure 6:**
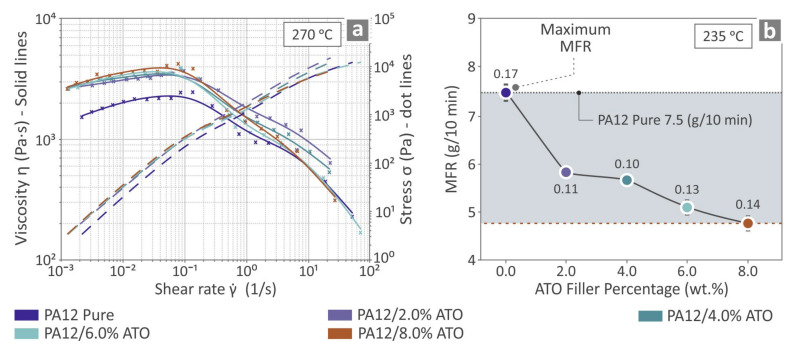
Referring to the PA12 pure and the PA12/ATO 2.0–8.0 wt.% composites: (**a**) viscosity and stress vs. shear rate curves at 270 °C (indicative measurements by which the curves were compiled are marked in the graph with an “x”) and (**b**) MFR vs. ATO quantity of filler at 235 °C. The numbers in the dots are the deviations calculated for each case.

The information provided in Figure 6 pertains to rheological examination tests. Figure 6a shows the derived data regarding viscosity versus shear rate, as well as stress versus shear rate, for the unfilled PA12 thermoplastic and the compounds of PA12/ATO 2.0–8.0 wt.%, at 270 °C. Figure 6b presents the calculated MFR for PA12 pure and all the PA12/ATO composites at 235 °C. By observing the viscosity-related information, it can be concluded that, as stress increases, the viscosity decreases. However, increasing the filler content in the compounds resulted in a reduction in the Melt Flow Rate.

### 3.4. Quality Assurance of the Prepared Filaments

**Figure 7:**
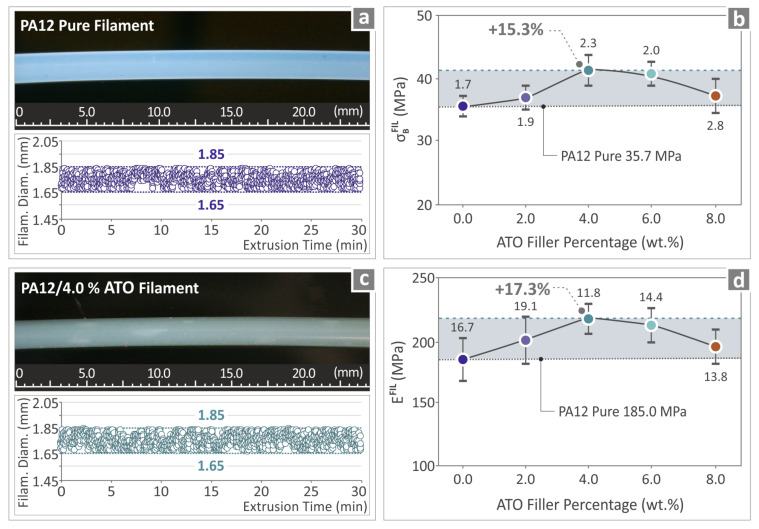
(**a**) Microscope picture of the extruded PA12 pure filament and the results derived from its diameter monitoring; (**b**) tensile strength results regarding PA12 pure and all PA12/ATO composite filaments; (**c**) Microscope image of the extruded PA12/ATO 4.0 wt.% filament and the results derived from its diameter monitoring; (**d**) tensile modulus of elasticity results regarding PA12 pure and the PA12/ATO nanocomposite filaments. Each loading is presented with a different color in the graphs, hence the different colors in the dots.

Figure 7 depicts the assessment of the prepared filaments’ quality and mechanical properties. In particular, Figure 7a,c depict the PA12 pure and PA12/ATO 4.0 wt.% filaments. These were of high quality, without defects. Additionally, the results from diameter monitoring indicate that the diameter values were steady in the range of 1.65 mm–1.85 mm, with the extrusion settings used. Figure 7b,d show the results of the mechanical test of PA12 pure and all PA12/ATO nanocomposites’ filaments. In Figure 7b, the strength derived in the tensile tests of the PA12/ATO 4.0 wt.% was found to be 15.3% above PA12 pure. In Figure 7d, the tensile modulus of elasticity calculated in the tensile tests for the PA12/ATO 4.0 wt.% is presented, and it was above PA12 pure by 17.3%.

### 3.5. Mechanical Properties of the Produced Specimens

**Figure 8:**
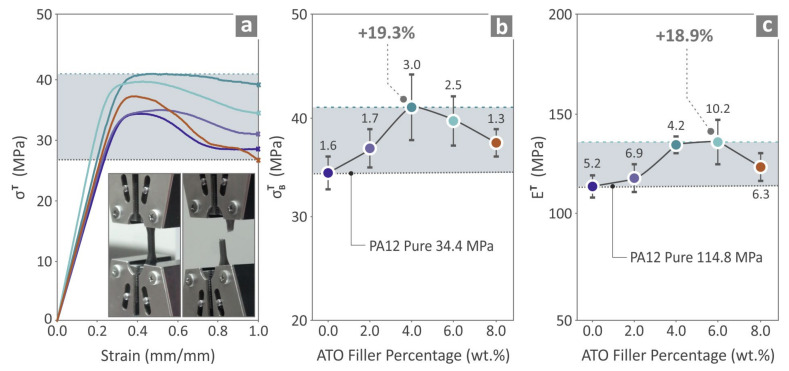
Results from the tensile test for the unfilled PA12 and PA12/ATO 2.0 wt.%–8.0 wt.% composites, namely (**a**) stress vs. strain plots (tensile test) and two images captured in the course of the experimental procedure, tensile (**b**) strength values (mean and deviation), and (**c**) modulus of elasticity (mean and deviation).

The results derived for the mechanical properties of the specimens are shown in the following three figures. In Figure 8, the tensile properties are presented. Figure 8a depicts the graphs of tensile stress vs. strain and two images captured from the experimental procedure. Figure 8b shows the tensile strength results, indicating that the composite with the highest performance is PA12/ATO 4.0 wt.%, which is 19.3% above the tensile strength of PA12 pure. Figure 8c shows the tensile modulus of elasticity values (mean and deviation), in which PA12/ATO 6.0 wt.% was the one having the most improved behavior, 18.9% higher in relation to PA12 pure.

**Figure 9:**
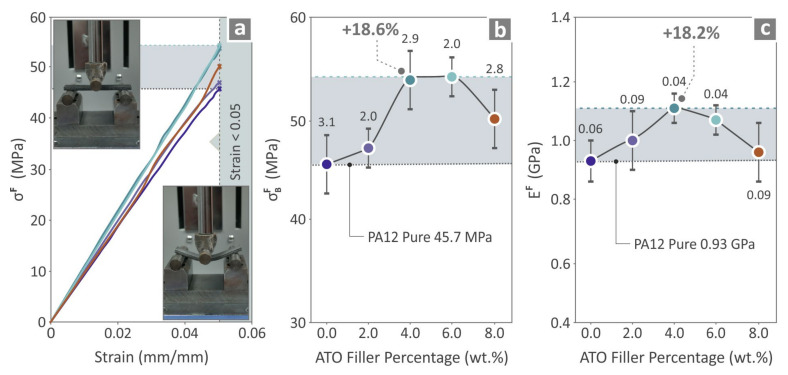
Findings from the flexural experimental process of the PA12 pure and PA12/ATO 2.0 wt.%–8.0 wt.% composites (**a**) flexural stress to strain graphs and two images captured during the testing, flexural (**b**) strength values (mean and deviation), and (**c**) modulus of elasticity (mean and deviation).

Figure 9 presents the mechanical properties derived from the flexural tests of the unfilled PA12 and all the PA12/ATO composites. Figure 9a depicts the flexural stress on the strain graphs and two depictions from flexural testing of the samples. Figure 9b shows the flexural strength results and highlights PA12/ATO 4.0 wt.%, as the composite is 18.6% higher than PA12 pure. In Figure 9c, the flexural modulus of elasticity results are shown, and the PA12/ATO 4.0 wt.% composite is distinguished by being 18.2% higher than PA12 pure.

**Figure 10:**
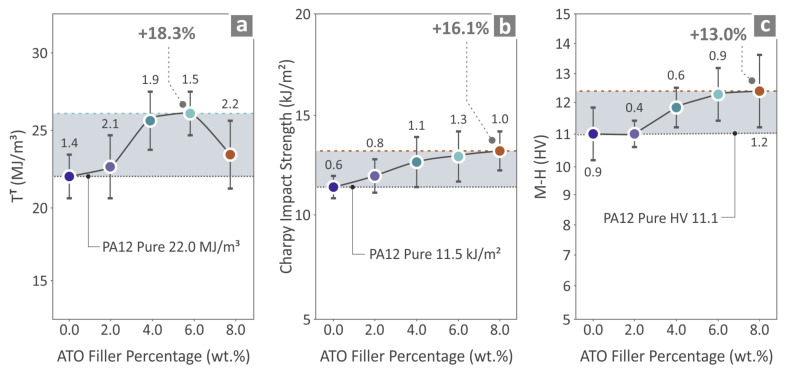
Results from the (**a**) tensile toughness, (**b**) strength in the impact test (Charpy), and (**c**) Vickers microhardness of the unfilled PA12 pure and PA12/ATO 2.0–8.0 wt.% composite specimens.

Figure 10a depicts the tensile toughness (calculated as the integral derived from the stress-to-strain graph) outcome for the unfilled PA12 and PA12/ATO 2.0–8.0 wt.% composites, where the PA12/ATO 6.0 wt.% has the most increased value over pure PA12 (18.3%). Figure 10b shows the Charpy impact strength results, where PA12/ATO 8.0 wt.% sample was found to be 16.1% higher than PA12 pure. In Figure 10c, the M–H results are shown, indicating that the PA12/ATO 8.0 wt.% composite was over PA12 pure by 13.0%. Both the impact strength and the M–H measurements constantly increased with the increase in the ATO nanoparticle percentage in the nanocomposites.

### 3.6. μ-Computed Tomography Results

**Figure 11:**
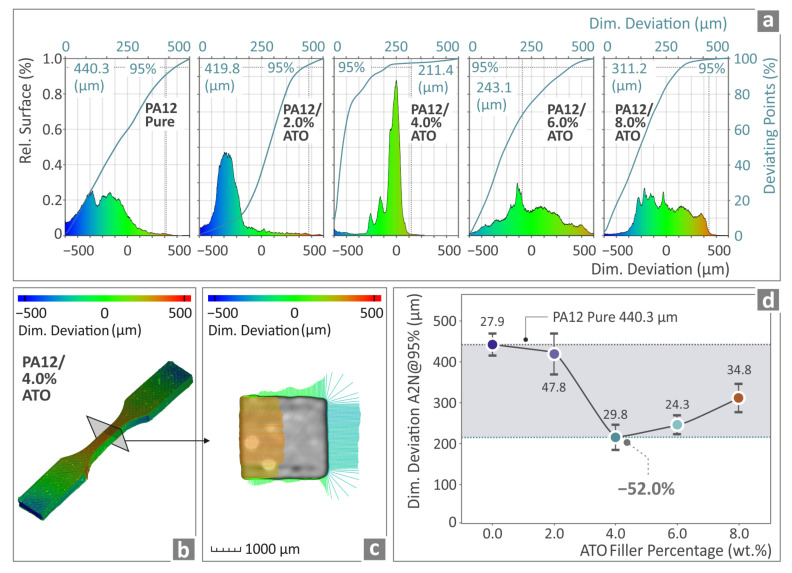
(**a**) The derived information regarding PA12 pure and the total amount of PA12/ATO composite specimens’ μ-CT in relation to their dimensional deviation; (**b**,**c**) PA12/ATO 4.0 wt.% tensile examples’ geometrical deviation presented utilizing a color-coded MAP; (**d**) A2N at 95% of PA12 pure and the total amount of PA12/ATO composites.

**Figure 12:**
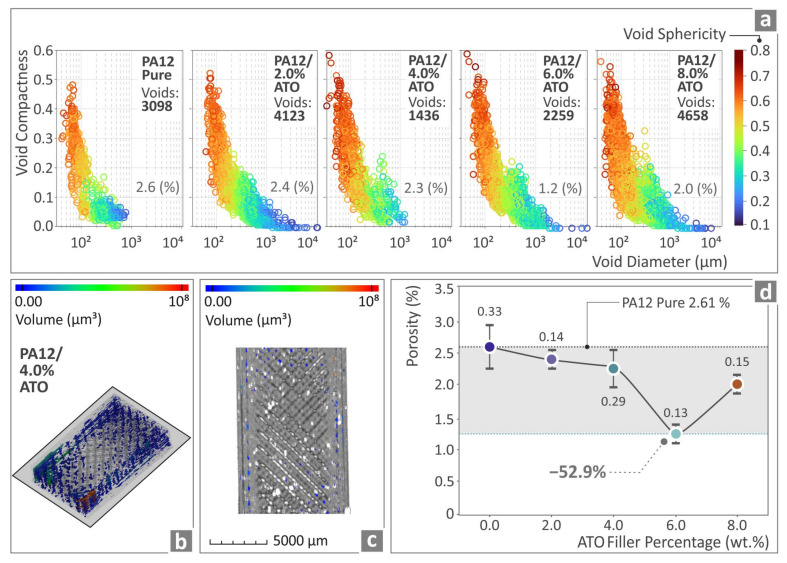
(**a**) The results regarding PA12 pure and all the PA12/ATO composite specimens’ μ-CT in relation to their porosity; (**b**,**c**) PA12/ATO 6.0 wt.% tensile specimen’s visual representation of porosity through color-coded MAP; (**d**) porosity of PA12 pure and all of the PA12/ATO nanocomposites.

Figure 11 and Figure 12 show information regarding the dimensional accuracy and voids, respectively, of the manufactured specimens. Figure 11a shows the μ-CT results for PA12 pure and all PA12/ATO 2.0 wt.%–8.0 wt.% composites in graphs for the deviation of the geometry from the nominal values. In Figure 11b,c, color-coding mapping was used to achieve PA12/ATO 4.0 wt.% tensile specimen comparison of the structural deviations and data of the created CAD model. Figure 11d shows the A2N at 95% (5% of the measurements having excessive values were removed to increase the accuracy of the finding) values of PA12 pure and the total amount of the PA12/ATO nanocomposites’ samples. The lowest value was detected for PA12/ATO 4.0 wt.%, which was 52.0% lower than pure PA12. However, all filler percentages provided samples with better dimensional deviations than those of the PA12 pure. Overall, the introduction of the ATO nanoparticles increased the accuracy of the geometry of the samples (vs. the control sample of the unfilled PA12).

In Figure 12a, the μ-CT scanning data related to the porosity of PA12 pure and PA12/ATO 2.0 wt.%–8.0 wt.% composites are shown in graphs regarding how compact and spherical the voids are with respect to their diameter. Figure 12b,c show how color-coding mapping is utilized for the presentation of the PA12/ATO 4.0 wt.% sample’s void distribution and volumes. Figure 12d depicts the porosity of the unfilled PA12 and all the PA12/ATO composites’ samples. The sample of PA12/ATO 6.0 wt.% was found to have porosity decreased by 52.9% compared with pure PA12, while the rest of the composites also presented improved behavior in relation to pure PA12. Although the number of voids is higher than the pure PA12 sample in the case of the nanocomposites having 2.0 and 8.0 wt.% ATO content, the porosity percentage is lower in all nanocomposites compared with the pure PA12 polymer.

### 3.7. Morphological and Chemical Analysis Results of the 3D-P Samples

**Figure 13:**
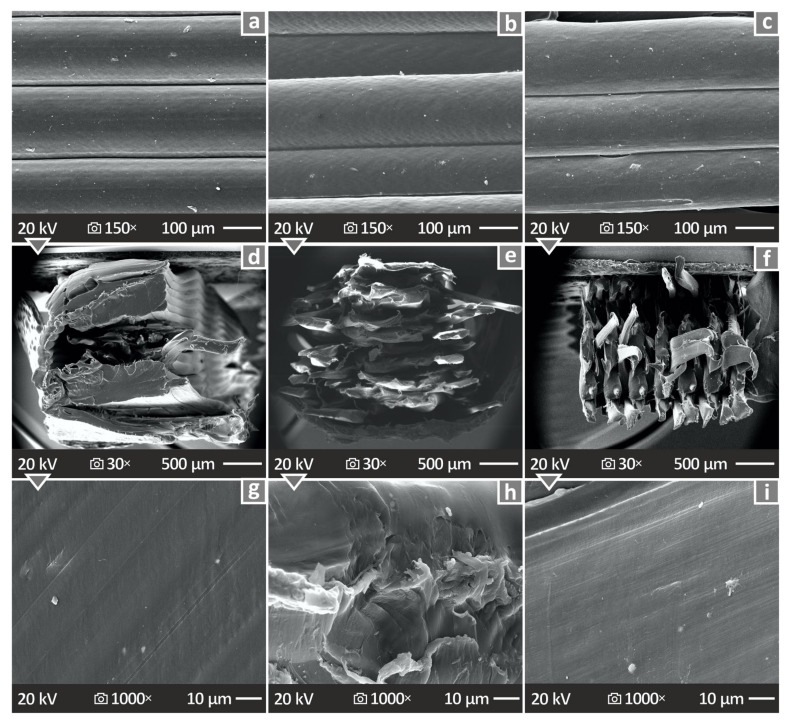
SEM pictures of (**a**–**c**) PA12/ATO 2.0, 4.0, and 8.0 wt.% specimens’ vertical surface magnified in 150×, (**d**–**f**) PA12/ATO 2.0, 4.0, and 8.0 wt.% specimens’ fracture cross-section magnified 30×, and (**g**–**i**) PA12/ATO 2.0, 4.0, and 8.0 wt.% specimens’ fracture cross-section magnified 1000×.

**Figure 14:**
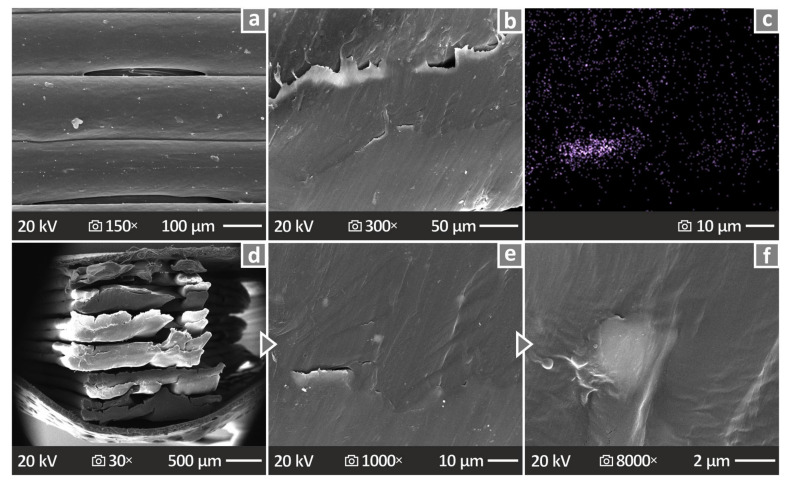
(**a**,**b**) PA12/ATO 6.0 wt.% vertical surface SEM images at 150× and 300× magnifications, correspondingly, (**c**) EDS mapping image of PA12/ATO 6.0 wt.%, (**d**–**f**) PA12/ATO 6.0 wt.% fracture surface SEM images magnified 30× and 1000× and 8000×.

Figure 13 and Figure 14 present the illustrations captured through SEM investigation of the MEX 3D-P specimens. Figure 13a–c show the side surface pictures of PA12/ATO 2.0, 4.0 and 8.0 wt.% specimens, respectively, magnified by 150×. Figure 13d–f depict the fracture cross-section pictures of PA12/ATO 2.0, 4.0, and 8.0 wt.% specimens, respectively, magnified by 30×, while Figure 13g–i show the same samples, but at 1000× magnification. The side surfaces exhibited significant layering without defects or voids. The fracture surface images revealed brittle behavior (no visible high deformation was observed). In the higher-loaded specimens, the samples seem to have collapsed during the breakage in the tensile test.

Figure 14a,b illustrate the lateral surface pictures of the PA12/ATO 6.0 wt.% sample at 150× and 300× magnifications, respectively. Figure 14c illustrates an EDS mapping for the Sn element of the same sample. Figure 14d–f present the SEM illustrations of the PA12/ATO 6.0 wt.% sample’s fracture surface at several magnifications, namely, 30×, 1000×, and 8000×, respectively. The side surfaces presented well-distributed layering, except for a few voids. Again, the fracture surface was found to be brittle. Closer magnification did not reveal any voids, pores, or defects.

## 4. Discussion

The mechanical test’s experimental results revealed an obvious improvement in all the composite samples, verifying ATO’s effectiveness in reinforcing the PA12 polymer in MEX 3D printing, which was the main hypothesis of the research. For PA12/ATO 4.0 wt.%, several mechanical properties, i.e., tensile and flexural strength and flexural modulus of elasticity, revealed the greatest improvement. However, the PA12/ATO 6.0 wt.% also presented improved behavior in its tensile toughness and modulus of elasticity. Additionally, PA12/ATO 8.0 wt.% results of Charpy impact strength and M–H revealed the greatest values among the samples assessed. The specimens were also subjected to μCT scanning to examine their dimensional deviations and porosities. These results presented remarkable performance, as they decreased by 52.0% (PA12/ATO 4.0 wt.%) and 52.9% (PA12/ATO 6.0 wt.%), respectively (vs. the unfilled PA12). The SEM pictures of the specimens presented very good layering by inspecting the side surfaces, whereas the fracture surfaces were characterized by brittle behavior.

**Figure 15:**
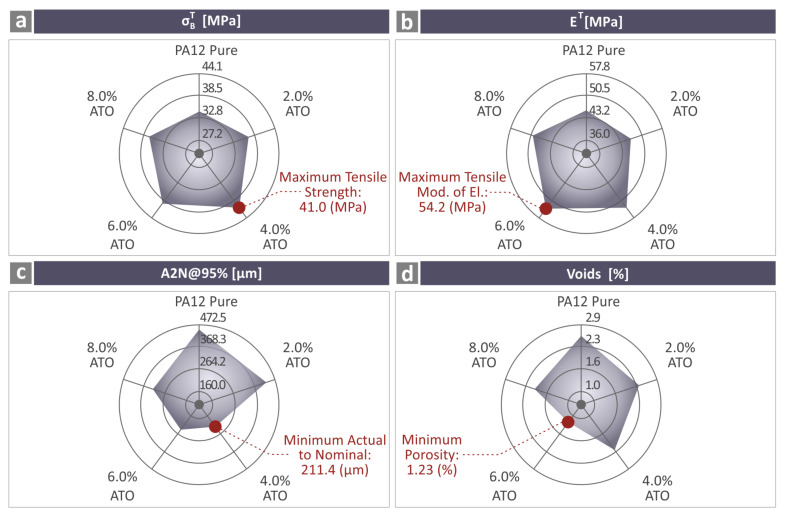
Summarization of the information from the tests on the PA12 pure and all PA12/ATO specimens in four different spider graphs, namely tensile (**a**) strength, (**b**) modulus of elasticity, (**c**) A2N at 95%, and (**d**) void percentage.

Overall, all the nanocomposite samples exhibited improved properties compared with those of the pure PA12 samples. The results are summarized in the spider-designed graphs in Figure 15. Figure 15a presents the tensile strength findings of the unfilled PA12 and all the PA12/ATO nanocomposites, while the maximum value reported is highlighted with a red dot in each graph. The values derived for the tensile modulus of elasticity are presented in Figure 15b. Figure 15c,d show the A2N dimensional deviation and void results, respectively.

One critical aspect when nanocomposites are dispersed is the filler in the composite’s matrix [89]. Possible aggregate might result in a negative impact on the mechanical performance of the samples [89,90]. A specially designed extruder for material mixing was used to ensure the effective dispersion of ATO nanoparticles within the PA12 matrix. To verify the good dispersion of the nanoparticles, SEM was used, which is a common practice in the literature [91,92,93]. By observing the fracture surfaces, no particle clustering was located. Additionally, EDS revealed a similar finding in the observation areas, with a rather uniform dispersion of the particles, judging from the ATO’s element distribution derived through EDS mapping. Finally, the mechanical tests on the samples did not exhibit significant deviations in the results, which is an additional indication of a uniform composition in the examples tested.

Regarding the selection of the filler content in the composites, it should be noted that the mechanical tensile and flexural properties started to decline when the ATO filler content exceeded 4.0 wt.%. Still, two additional composites were tested, with the ATO filler content reaching 8.0 wt.% in the highest loading. When the mechanical properties started to decline with the increase in ATO content in the composites, testing only one additional filler percentage could not be enough in some cases. The mechanical properties can decline for some reason other than the saturation of the filler in the matrix, and then start to increase again at higher filler loadings. With the further increase in filler content, the properties further decreased, as was the case in the current study for both the tensile and flexural test results. This decrease in the mechanical properties can be safely assumed to be due to the saturation of the filler in the matrix. That is why the authors tested the highest filler percentage in the matrix as well, to be on the safe side regarding the reason that the mechanical properties decreased at higher filler content in the composites. Regarding the mechanical properties, the tensile and flexural strengths increased first and then decreased, but the impact strength increased monotonically. Different responses are expected in quasi-static and impact stress conditions. ATO seems to enhance the intense impact energy-absorbing performance. The impact test measures the energy a polymer can absorb before fracturing. A low energy absorption indicates the material is fragile and highly sensitive to impact forces. Conversely, high energy absorption suggests the material is soft, deformable, and possesses high toughness, allowing it to withstand greater impact forces. The influence of adding nanoparticles on energy absorption varies across different composites and depends on the matrix. Therefore, the high energy absorption of PA12 contributed to this effect in this case [94].

For the thermal properties, it was found that the addition of the ATO nanoparticles did not affect the thermal stability of the PA12 thermoplastic. Additionally, TGA proved that the extrusion temperatures utilized do not degrade the nanocomposites, which could affect the mechanical properties of the composites as derived from the test results [95,96,97]. DSC measurements showed that the PA12/ATO 4 wt.% nanocomposite had the lowest Tm among the examples tested. This nanocomposite had the highest mechanical response at the same time. So, there is a possible correlation between the Tm and the mechanical response, which needs further investigation and exceeds the scope of the research.

The rheology measurements revealed a constant decrease in the MFR values while increasing the ATO percentage existing in the nanocomposites. This would suggest that the 3D printing parameters require adjustment for each nanocomposite to achieve better quality and performance of 3D printed items, as the rheology affects these aspects [98]. Various SEM illustrations on the side regions of the examples did not reveal significant differences between the specimens, as would be expected. The MFR reduction was expected to result in reduced fusion quality between the layers and non-uniform-thickness layers. This was not the case; still, the different viscosity, MFR, and Tm values in the nanocomposites are estimated to impact the outcome of the 3D printing formation.

The introduction of the ATO filler in nanoparticle form in the PA12 thermoplastic overall improved the two quality metrics evaluated, i.e., the spatial characteristics and the porosity of the 3D printed materials. The PA12/ATO 4.0 wt.% nanocomposite specimens had the most accurate geometry compared with the nominal one among the examples measured. This shows that higher-built quality can positively impact mechanical performance as well. Regarding porosity, literature reports that reduced porosity improves mechanical performance [99,100]. The lowest porosity was found in the PA12/ATO 6.0 wt.% nanocomposite specimens, but the porosity of the PA12/ATO 4.0 wt.% nanocomposite specimens was very close. This agrees with the literature that a decrease in porosity in the 3D printing structure can improve mechanical performance. Still, the increased mechanical performance of the PA12/ATO 4.0 wt.% nanocomposite specimens can be attributed mainly to the presence of the nanoparticles, but other factors, such as the quality metrics presented, can have an impact on this outcome.

The bibliography search did not reveal any similar nanocomposites to correlate with the findings, especially for the AM technology. To evaluate the results in the literature, the mechanical test results were compared with the respective results in other composites prepared with different polymeric matrix materials. A similar thermomechanical extrusion process was employed for their preparation and the MEX 3D printing method as well. So, the results were on 3D-printed samples, and the impact of the 3D printing process on the strength was considered. The comparison is shown in the following Table 3. As shown, the reinforcement effect of the ATO nanocomposites was greater in the PA12 polymer studied herein compared with the other polymeric matrices. The differences are not significant. The lowest reinforcement was reported for the PP polymer. Still, the differences justify the need for individual research for each polymeric material, as the interaction of the nanoparticles with the matrix and the processability differ.

## 5. Conclusions

In this study, PA12/ATO composites with different filler percentages (2.0 wt.%–8.0 wt.%, with a 2.0 step) were prepared to investigate the reinforcement capacity of ATO in this specific matrix (PA12) following a process suitable to the MEX 3D printing technique. The raw materials were turned into mixtures, which were fed into a filament extruder, and the as-made filaments were subsequently driven into a three-dimensional printer for the manufacturing of the specimens. All the specimens and filaments were tested for their mechanical properties, and the results were correlated to those of the unfilled PA12 examples. Thermal, rheological, structural, and morphological investigations have also been conducted.

The derived results indicate an enhancement in all the composites in comparison with the behavior of the PA12 pure samples. The composites distinguished by their reinforcing behavior were PA12/ATO 4.0 wt.%, PA12/ATO 6.0 wt.%, and PA12/ATO 8.0 wt.%. SEM images were captured, while the μ-CT results clarified the significant influence of the ATO filler in the case of 4.0 wt.% as to the dimensional deviation and the 6.0 wt.% regarding the porosity. The key findings are as follows:

1. The ATO 4.0 wt.% content is considered the optimum filler content among the PA12/ATO nanocomposites prepared, with 19.3% and 18.6% increases in the tensile and flexural strength, respectively (vs. the control sample of the unfilled PA12).
2. The thermal invariance of the PA12 polymer was maintained and somewhat enhanced by the ATO nanoparticles’ introduction in the matrix.
3. The viscosity of the nanocomposites was slightly increased compared with the control sample (PA12 pure), while MFR constantly decreased while increasing the ATO content in the nanocomposites.
4. The ATO nanoparticle’s introduction increased the quality metrics of the 3D-printed specimens. The dimensional deviation was improved (decreased), and the percentage of voids in the 3D printed structure was decreased, especially in the PA12/ATO 6 wt.% nanocomposite.

Further investigations could be conducted to further optimize the sets of printing parameters and achieve maximum performance from the composites. This was not implemented herein for comparison purposes.

## Figures and Tables

**Table 1:** Major Raman peak discrepancies of PA12/ATO composites from PA12/Pure.

1000	Drop	Strong Decrease
1062	Rise	Medium increase
1106	Rise	Strong increase
1294	Rise	Strong increase
1435	Rise	Strong increase
1581	Drop	Medium decrease
1600	Drop	Medium decrease
2835	Gradual drop	Small decrease
2849	Rise	Strong increase
2883	Rise	Strong increase
2931	Rise	Medium increase
3052	Drop	Significant decrease

**Table 2:** Initial Decomposition Temperature (IDT) at 10% and 50% mass loss.

IDT at 10% (°C)	IDT at 50% (°C)
427.15	459.61
431.71	464.06
433.66	466.11
430.26	462.43
434.48	469.13
433.45	468.20

**Table 3:** ATO reinforcement efficacy in different matrices for nanocomposites prepared with a thermomechanical extrusion process for MEX 3D-P.

Increase (%)	Current	PP [70]	PETG [71]	ABS [62]
Tensile strength	19.3	7.0	21.0	~11.0
Flexural strength	18.6	8.0	14.4	~10.0
Impact strength	16.1	28.0	18.8	-
microhardness	13.0	5.0	25.1	-

## Data Availability

The raw/processed data required to reproduce these findings cannot be shared because of technical or time limitations.

## Supplementary Materials

The following supporting information can be downloaded at: https://www.mdpi.com/article/10.3390/nano14151285/s1, Table S1. Major Raman peaks of PA12 pure identified and their related assignments.

## References

[1] Mota C., Puppi D., Chiellini F., Chiellini E. (2015). Additive Manufacturing Techniques for the Production of Tissue Engineering Constructs. J. Tissue Eng. Regen. Med..

[2] Seo H., Heo S.G., Lee H., Yoon H. (2017). Preparation of PEG Materials for Constructing Complex Structures by Stereolithographic 3D Printing. RSC Adv..

[3] Waldbaur A., Rapp H., Länge K., Rapp B.E. (2011). Let There Be Chip—Towards Rapid Prototyping of Microfluidic Devices: One-Step Manufacturing Processes. Anal. Methods.

[4] Waheed S., Cabot J.M., Macdonald N.P., Lewis T., Guijt R.M., Paull B., Breadmore M.C. (2016). 3D Printed Microfluidic Devices: Enablers and Barriers. Lab Chip.

[5] Sachs E.M., Haggerty J.S., Cima M.J., Williams P.A. (1993). Three-Dimensional Printing Techniques. U.S. Patent.

[6] Hutmacher D.W., Sittinger M., Risbud M.V. (2004). Scaffold-Based Tissue Engineering: Rationale for Computer-Aided Design and Solid Free-Form Fabrication Systems. Trends Biotechnol..

[7] Beaman J.J., Barlow J.W., Bourell D.L., Crawford R.H., Marcus H.L., McAlea K.P. (1997). Solid Freeform Fabrication: A New Direction in Manufacturing with Research and Applications in Thermal Laser Processing.

[8] Comb J.W., Priedeman W.R., Turley P.W. FDM^®^ Technology Process Improvements. Proceedings of the 1994 International Solid Freeform Fabrication Symposium.

[9] Mazumder J., Schifferer A., Choi J. (1999). Direct Materials Deposition: Designed Macro and Microstructure. Mater. Res. Innov..

[10] Jacobs P.F. (1992). Rapid Prototyping & Manufacturing: Fundamentals of Stereolithography.

[11] Feygin M., Hsieh B. Laminaled Objecf Manufacturing (LOM): A Simpler Process. Proceedings of the 1991 International Solid Freeform Fabrication Symposium.

[12] Jain V.K. (2008). Advanced (Non-Traditional) Machining Processes. Machining.

[13] Ambrosi A., Pumera M. (2016). 3D-Printing Technologies for Electrochemical Applications. Chem. Soc. Rev..

[14] Thomas C.L., Gaffney T.M., Kaza S., Lee C.H. (1999). Rapid Prototyping of Large Scale Aerospace Structures. Proceedings of the 1996 IEEE Aerospace Applications Conference: Proceedings.

[15] Bacciaglia A., Ceruti A., Liverani A. (2022). Towards Large Parts Manufacturing in Additive Technologies for Aerospace and Automotive Applications. Procedia Comput. Sci..

[16] Mohanavel V., Ashraff Ali K.S., Ranganathan K., Allen Jeffrey J., Ravikumar M.M., Rajkumar S. (2021). The Roles and Applications of Additive Manufacturing in the Aerospace and Automobile Sector. Mater. Today Proc..

[17] Najmon J.C., Raeisi S., Tovar A. (2019). Review of Additive Manufacturing Technologies and Applications in the Aerospace Industry. Additive Manufacturing for the Aerospace Industry.

[18] Tepylo N., Huang X., Patnaik P.C. (2019). Laser-Based Additive Manufacturing Technologies for Aerospace Applications. Adv. Eng. Mater..

[19] de Leon A.C., Chen Q., Palaganas N.B., Palaganas J.O., Manapat J., Advincula R.C. (2016). High Performance Polymer Nanocomposites for Additive Manufacturing Applications. React. Funct. Polym..

[20] Campbell I., Bourell D., Gibson I. (2012). Additive Manufacturing: Rapid Prototyping Comes of Age. Rapid Prototyp. J..

[21] Ding D., Shen C., Pan Z., Cuiuri D., Li H., Larkin N., van Duin S. (2016). Towards an Automated Robotic Arc-Welding-Based Additive Manufacturing System from CAD to Finished Part. Comput. Aided Des..

[22] Raja V., Zhang S., Garside J., Ryall C., Wimpenny D. (2006). Rapid and Cost-Effective Manufacturing of High-Integrity Aerospace Components. Int. J. Adv. Manuf. Technol..

[23] Song Y., Yan Y., Zhang R., Xu D., Wang F. (2002). Manufacture of the Die of an Automobile Deck Part Based on Rapid Prototyping and Rapid Tooling Technology. J. Mater. Process Technol..

[24] Vasco J.C. (2021). Additive Manufacturing for the Automotive Industry. Additive Manufacturing.

[25] Dhakshyani R., Nukman Y., Abu Osman N., Vijay C. (2011). Preliminary Report: Rapid Prototyping Models for Dysplastic Hip Surgery. Open Med..

[26] Liu Q., Leu M.C., Schmitt S.M. (2006). Rapid Prototyping in Dentistry: Technology and Application. Int. J. Adv. Manuf. Technol..

[27] Petzold R., Zeilhofer H.-F., Kalender W.A. (1999). Rapid Prototyping Technology in Medicine—Basics and Applications. Comput. Med. Imaging Graph..

[28] Mognol P., Lepicart D., Perry N. (2006). Rapid Prototyping: Energy and Environment in the Spotlight. Rapid Prototyp. J..

[29] Liu Z., Zhang M., Bhandari B., Wang Y. (2017). 3D Printing: Printing Precision and Application in Food Sector. Trends Food Sci. Technol..

[30] Luo Y., Ji Z., Leu M.C., Caudill R. (1999). Environmental Performance Analysis of Solid Freedom Fabrication Processes. Proceedings of the 1999 IEEE International Symposium on Electronics and the Environment (Cat. No.99CH36357).

[31] Telenko C., Conner Seepersad C. (2012). A Comparison of the Energy Efficiency of Selective Laser Sintering and Injection Molding of Nylon Parts. Rapid Prototyp. J..

[32] Abdulhameed O., Al-Ahmari A., Ameen W., Mian S.H. (2019). Additive Manufacturing: Challenges, Trends, and Applications. Adv. Mech. Eng..

[33] Sun C., Wang Y., McMurtrey M.D., Jerred N.D., Liou F., Li J. (2021). Additive Manufacturing for Energy: A Review. Appl. Energy.

[34] Ning F., Cong W., Hu Z., Huang K. (2017). Additive Manufacturing of Thermoplastic Matrix Composites Using Fused Deposition Modeling: A Comparison of Two Reinforcements. J. Compos. Mater..

[35] Murphy C.A., Collins M.N. (2018). Microcrystalline Cellulose Reinforced Polylactic Acid Biocomposite Filaments for 3D Printing. Polym. Compos..

[36] Kam M., Saruhan H., Ipekci A. (2018). Investigation the Effects of 3D Printer System Vibrations on Mechanical Properties of the Printed Products. Sigma J. Eng. Nat. Sci..

[37] Kam M., İpekçi A., Saruhan H. (2017). Investigation of 3d Printing Filling Structures Effect on Mechanical Properties and Surface Roughness of PET-G Material Products. Gaziosmanpaşa Bilimsel Araştırma Derg..

[38] Petousis M., Vidakis N., Mountakis N., Papadakis V., Tzounis L. (2022). Three-Dimensional Printed Polyamide 12 (PA12) and Polylactic Acid (PLA) Alumina (Al_2_O_3_) Nanocomposites with Significantly Enhanced Tensile, Flexural, and Impact Properties. Nanomaterials.

[39] Tyuftin A.A., Kerry J.P. (2020). Review of Surface Treatment Methods for Polyamide Films for Potential Application as Smart Packaging Materials: Surface Structure, Antimicrobial and Spectral Properties. Food Packag. Shelf Life.

[40] Shao S., Zeng F., Long L., Zhu X., Peng L.E., Wang F., Yang Z., Tang C.Y. (2022). Nanofiltration Membranes with Crumpled Polyamide Films: A Critical Review on Mechanisms, Performances, and Environmental Applications. Environ. Sci. Technol..

[41] De Schoenmaker B., Van der Heijden S., De Baere I., Van Paepegem W., De Clerck K. (2013). Effect of Electrospun Polyamide 6 Nanofibres on the Mechanical Properties of a Glass Fibre/Epoxy Composite. Polym. Test..

[42] Supaphol P., Mit-Uppatham C., Nithitanakul M. (2005). Ultrafine Electrospun Polyamide-6 Fibers: Effect of Emitting Electrode Polarity on Morphology and Average Fiber Diameter. J. Polym. Sci. B Polym. Phys..

[43] Heikkilä P., Harlin A. (2008). Parameter Study of Electrospinning of Polyamide-6. Eur. Polym. J..

[44] Durkan R., Ayaz E.A., Bagis B., Gurbuz A., Ozturk N., Korkmaz F.M. (2013). Comparative Effects of Denture Cleansers on Physical Properties of Polyamide and Polymethyl Methacrylate Base Polymers. Dent. Mater. J..

[45] Soygun K., Bolayir G., Boztug A. (2013). Mechanical and Thermal Properties of Polyamide versus Reinforced PMMA Denture Base Materials. J. Adv. Prosthodont..

[46] Kim J.H., Choe H.C., Son M.K. (2014). Evaluation of Adhesion of Reline Resins to the Thermoplastic Denture Base Resin for Non-Metal Clasp Denture. Dent. Mater. J..

[47] Vidakis N., Petousis M., Ntintakis I., David C., Sagris D., Mountakis N., Moutsopoulou A. (2024). Quantitative Insight into the Compressive Strain Rate Sensitivity of Polylactic Acid, Acrylonitrile Butadiene Styrene, Polyamide 12, and Polypropylene in Material Extrusion Additive Manufacturing. J. Dyn. Behav. Mater..

[48] Balabanov S.V., Makogon A.I., Sychev M.M., Evstratov A.A., Regazzi A., Lopez-Cuesta J.M. (2020). 3D Printing and Mechanical Properties of Polyamide Products with Schwartz Primitive Topology. Tech. Phys..

[49] Petousis M., Spiridaki M., Mountakis N., Moutsopoulou A., Maravelakis E., Vidakis N. (2024). Box-Behnken Modeling to Optimize the Engineering Response and the Energy Expenditure in Material Extrusion Additive Manufacturing of Short Carbon Fiber Reinforced Polyamide 6. Int. J. Adv. Manuf. Technol..

[50] Chowdhury M.R., Steffes J., Huey B.D., McCutcheon J.R. (2018). 3D Printed Polyamide Membranes for Desalination. Science.

[51] Zhang X., Fan W., Liu T. (2020). Fused Deposition Modeling 3D Printing of Polyamide-Based Composites and Its Applications. Compos. Commun..

[52] Vidakis N., Petousis M., Velidakis E., Mountakis N., Grammatikos S.A., Tzounis L. (2023). Multi-Functional Medical Grade Polyamide12/Carbon Black Nanocomposites in Material Extrusion 3D Printing. Compos. Struct..

[53] Vidakis N., Petousis M., Michailidis N., Grammatikos S., David C.N., Mountakis N., Argyros A., Boura O. (2022). Development and Optimization of Medical-Grade MultiFunctional Polyamide 12-Cuprous Oxide Nanocomposites with Superior Mechanical and Antibacterial Properties for Cost-Effective 3D Printing. Nanomaterials.

[54] Vidakis N., Petousis M., Mountakis N., Korlos A., Papadakis V., Moutsopoulou A. (2022). Trilateral Multi-Functional Polyamide 12 Nanocomposites with Binary Inclusions for Medical Grade Material Extrusion 3D Printing: The Effect of Titanium Nitride in Mechanical Reinforcement and Copper/Cuprous Oxide as Antibacterial Agents. J. Funct. Biomater..

[55] Vidakis N., Petousis M., Michailidis N., Mountakis N., Papadakis V., Argyros A., Charou C. (2023). Polyethylene Glycol and Polyvinylpyrrolidone Reduction Agents for Medical Grade Polyamide 12/Silver Nanocomposites Development for Material Extrusion 3D Printing: Rheological, Thermomechanical, and Biocidal Performance. React. Funct. Polym..

[56] Vidakis N., Petousis M., Michailidis N., Mountakis N., Papadakis V., Argyros A., Charou C. (2023). Medical Grade Polyamide 12 Silver Nanoparticle Filaments Fabricated with In-Situ Reactive Reduction Melt-Extrusion: Rheological, Thermomechanical, and Bactericidal Performance in MEX 3D Printing. Appl. Nanosci..

[57] Hou Y., Panesar A. (2023). Effect of Manufacture-Induced Interfaces on the Tensile Properties of 3D Printed Polyamide and Short Carbon Fibre-Reinforced Polyamide Composites. Polymers.

[58] Peng Y., Wu Y., Li S., Wang K., Yao S., Liu Z., Garmestani H. (2020). Tailorable Rigidity and Energy-Absorption Capability of 3D Printed Continuous Carbon Fiber Reinforced Polyamide Composites. Compos. Sci. Technol..

[59] Beylergil B., Al-Nadhari A., Yildiz M. (2023). Optimization of Charpy-Impact Strength of 3D-Printed Carbon Fiber/Polyamide Composites by Taguchi Method. Polym. Compos..

[60] Egdell R.G., Flavell W.R., Tavener P. (1984). Antimony-Doped Tin(IV) Oxide: Surface Composition and Electronic Structure. J. Solid. State Chem..

[61] Hoflund G.B., Cox D.F., Woodson G.L., Laitinen H.A. (1981). Surface Characteristics of Antimony-Doped Tin Oxide Films. Thin Solid. Film..

[62] Vidakis N., Petousis M., Maniadi A., Koudoumas E., Liebscher M., Tzounis L. (2020). Mechanical Properties of 3D-Printed Acrylonitrile–Butadiene–Styrene TiO_2_ and ATO Nanocomposites. Polymers.

[63] Wu M., Zheng H., Li X., Yu S. (2020). Highly Transparent Low Resistance ATO/AgNWs/ATO Flexible Transparent Conductive Thin Films. Ceram. Int..

[64] Xiao Y., Tian W., Yu L., Chen M., Zheng X., Qin G. (2024). Tunable Optical Properties of ATO-CuO Hybrid Nanofluids and the Application as Spectral Beam Splitters. Energy.

[65] Yang Z., Zhang M., Zhao X., Guo Z., Zeb S., Jiang W., Liu T., Hu R., Jiang X. (2024). Ammonia Induced Strong LSPR Effect of Chain-like ATO Nanocrystals for Hyperspectral Selective Energy-Saving Window Applications. Chem. Eng. J..

[66] Chopra K.L., Major S., Pandya D.K. (1983). Transparent Conductors—A Status Review. Thin Solid. Films.

[67] Ginley D.S., Bright C. (2000). Transparent Conducting Oxides. MRS Bull..

[68] Gordon R.G. (2000). Criteria for Choosing Transparent Conductors. MRS Bull..

[69] Lewis B.G., Paine D.C. (2000). Applications and Processing of Transparent Conducting Oxides. MRS Bull..

[70] Vidakis N., Petousis M., Velidakis E., Mountakis N., Fischer-Griffiths P.E., Grammatikos S.A., Tzounis L. (2022). Mechanical Reinforcement Course of 3D Printed Polypropylene—Antimony Doped Tin Oxide Nanocomposites versus Filler Loading. Adv. Compos. Mater..

[71] Petousis M., Michailidis N., Saltas V., Papadakis V., Spiridaki M., Mountakis N., Argyros A., Valsamos J., Nasikas N.K., Vidakis N. (2024). Mechanical and Electrical Properties of Polyethylene Terephthalate Glycol/Antimony Tin Oxide Nanocomposites in Material Extrusion 3D Printing. Nanomaterials.

[72] Wang K., Xie G., Xiang J., Li T., Peng Y., Wang J., Zhang H. (2022). Materials Selection of 3D Printed Polyamide-Based Composites at Different Strain Rates: A Case Study of Automobile Front Bumpers. J. Manuf. Process.

[73] Zare Y., Rhee K.Y., Hui D. (2017). Influences of Nanoparticles Aggregation/Agglomeration on the Interfacial/Interphase and Tensile Properties of Nanocomposites. Compos. B Eng..

[74] Song Y., Zheng Q. (2016). Concepts and Conflicts in Nanoparticles Reinforcement to Polymers beyond Hydrodynamics. Prog. Mater. Sci..

[75] (2017). Standard Test Method for Tensile Properties of Plastics.

[76] (2016). Standard Test Methods for Flexural Properties of Unreinforced and Reinforced Plastics and Electrical Insulating Materials.

[77] (2004). Standard Test Method for Determining the Charpy Impact Resistance of Notched Specimens of Plastics.

[78] (2022). Standard Test Method for Microindentation Hardness of Materials.

[79] (2020). Standard Test Method for Melt Flow Rates of Thermoplastics by Extrusion Plastometer.

[80] Stuart B.H. (1996). Temperature Studies of Polycarbonate Using Fourier Transform Raman Spectroscopy. Polym. Bull..

[81] Makarem M., Lee C.M., Kafle K., Huang S., Chae I., Yang H., Kubicki J.D., Kim S.H. (2019). Probing Cellulose Structures with Vibrational Spectroscopy. Cellulose.

[82] Resta V., Quarta G., Lomascolo M., Maruccio L., Calcagnile L. (2015). Raman and Photoluminescence Spectroscopy of Polycarbonate Matrices Irradiated with Different Energy 28Si+ Ions. Vacuum.

[83] Zimmerer C., Matulaitiene I., Niaura G., Reuter U., Janke A., Boldt R., Sablinskas V., Steiner G. (2019). Nondestructive Characterization of the Polycarbonate-Octadecylamine Interface by Surface Enhanced Raman Spectroscopy. Polym. Test..

[84] Luiz B.K.M., Amboni R.D.M.C., Prates L.H.M., Roberto Bertolino J., Pires A.T.N. (2007). Influence of Drinks on Resin Composite: Evaluation of Degree of Cure and Color Change Parameters. Polym. Test..

[85] Gatin E., Iordache S.-M., Matei E., Luculescu C.-R., Iordache A.-M., Grigorescu C.E., Ilici R.R. (2022). Raman Spectroscopy as Spectral Tool for Assessing the Degree of Conversion after Curing of Two Resin-Based Materials Used in Restorative Dentistry. Diagnostics.

[86] Peris-Díaz M.D., Łydżba-Kopczyńska B., Sentandreu E. (2018). Raman Spectroscopy Coupled to Chemometrics to Discriminate Provenance and Geological Age of Amber. J. Raman Spectrosc..

[87] Liu X., Zou Y., Li W., Cao G., Chen W. (2006). Kinetics of Thermo-Oxidative and Thermal Degradation of Poly(d,l-Lactide) (PDLLA) at Processing Temperature. Polym. Degrad. Stab..

[88] Subramaniam M.P., Arunachalam G., Kandasamy R., Veluswamy P., Hiroya I. (2018). Effect of PH and Annealing Temperature on the Properties of Tin Oxide Nanoparticles Prepared by Sol–Gel Method. J. Mater. Sci. Mater. Electron..

[89] Shrestha S., Wang B., Dutta P. (2020). Nanoparticle Processing: Understanding and Controlling Aggregation. Adv. Colloid. Interface Sci..

[90] Wu Y., Wang C., Yang T. (2022). Aggregation of Nanoparticles and Their Effect on Mechanical Properties of Carbon Nanotube Networks. Comput. Mater. Sci..

[91] Venkateshaiah A., Padil V.V.T., Nagalakshmaiah M., Waclawek S., Černík M., Varma R.S. (2020). Microscopic Techniques for the Analysis of Micro and Nanostructures of Biopolymers and Their Derivatives. Polymers.

[92] Alqaheem Y., Alomair A.A. (2020). Microscopy and Spectroscopy Techniques for Characterization of Polymeric Membranes. Membranes.

[93] Soliman T.S., Vshivkov S.A., Elkalashy S.I. (2020). Structural, Linear and Nonlinear Optical Properties of Ni Nanoparticles–Polyvinyl Alcohol Nanocomposite Films for Optoelectronic Applications. Opt. Mater..

[94] Shan L., Tan C.Y., Shen X., Ramesh S., Zarei M.S., Kolahchi R., Hajmohammad M.H. (2023). The Effects of Nano-Additives on the Mechanical, Impact, Vibration, and Buckling/Post-Buckling Properties of Composites: A Review. J. Mater. Res. Technol..

[95] Da Costa H.M., Ramos V.D., de Oliveira M.G. (2007). Degradation of Polypropylene (PP) during Multiple Extrusions: Thermal Analysis, Mechanical Properties and Analysis of Variance. Polym. Test..

[96] Wu Z., Li F., Huang L., Shi Y., Jin X., Fang S., Chuang K., Lyon R.E., Harris F.W., Cheng S.Z.D. (2000). The Thermal Degradation Mechanism and Thermal Mechanical Properties of Two High Performance Heterocyclic Polymer Fibers. J. Therm. Anal. Calorim..

[97] Pliquet M., Rapeaux M., Delange F., Bussiere P.O., Therias S., Gardette J.L. (2021). Multiscale Analysis of the Thermal Degradation of Polyamide 6,6: Correlating Chemical Structure to Mechanical Properties. Polym. Degrad. Stab..

[98] Plummer C.J.G., Rodlert M., Bucaille J.-L., Grünbauer H.J.M., Månson J.-A.E. (2005). Correlating the Rheological and Mechanical Response of Polyurethane Nanocomposites Containing Hyperbranched Polymers. Polymer.

[99] Wang X., Zhao L., Fuh J.Y.H., Lee H.P. (2019). Effect of Porosity on Mechanical Properties of 3D Printed Polymers: Experiments and Micromechanical Modeling Based on X-ray Computed Tomography Analysis. Polymers.

[100] Al-Maharma A.Y., Patil S.P., Markert B. (2020). Effects of Porosity on the Mechanical Properties of Additively Manufactured Components: A Critical Review. Mater. Res. Express.

