# Supplementary material for: A Comprehensive Optimization Course of Antimony Tin Oxide Nanofiller Loading in Polyamide 12: Printability, Quality Assessment, and Engineering Response in Additive Manufacturing
Source: Nanomaterials (Basel). 2024 Jul 30;14(15):1285. doi: 10.3390/nano14151285 (PMC11314480; doi:10.3390/nano14151285)
Supplement: Supplementary file 1 [file nanomaterials-14-01285-s001.zip › nanomaterials-3128476-supplementary.pdf]

nanomaterials

MDPI

Article

A Comprehensive Optimization Course of Antimony Tin Oxide Nanofiller Loading in Polyamide 12: Printability, Quality Assessment, and Engineering Response in Additive Manufacturing

Nektarios K. Nasikas [1], Markos Petousis [2], Vassilis Papadakis [3,4], Apostolos Argyros [5,6], John Valsamos [2], Katerina Gkagkanatsiou [2], Dimitrios Sagris [7], Constantine David [7], Nikolaos Michailidis [5,6], Emmanuel Maravelakis [8] and Nectarios Vidakis [2,*]

1 Division of Mathematics and Engineering Sciences, Department of Military Sciences, Hellenic Army Academy, Vari, 16673 Athens, Attica, Greece
2 Department of Mechanical Engineering, Hellenic Mediterranean University, 71410 Heraklion, Greece
3 Department of Industrial Design and Production Engineering, University of West Attica, 12243 Athens, Greece
4 Institute of Electronic Structure and Laser, Foundation for Research and Technology-Hellas, N. Plastira 100m, 70013 Heraklion, Greece
5 Physical Metallurgy Laboratory, Mechanical Engineering Department, School of Engineering, Aristotle University of Thessaloniki, 54124 Thessaloniki, Greece
6 Centre for Research & Development of Advanced Materials (CERDAM), Center for Interdisciplinary Research and Innovation, Balkan Centre, Building B', 10th km Thessaloniki-Thermi Road, 57001 Thessaloniki, Greece
7 Department of Mechanical Engineering, International Hellenic University, Serres Campus, 62124 Serres, Greece
8 Department of Electronic Engineering, Hellenic Mediterranean University, 73133 Chania, Greece
* Correspondence: vidakis@hmu.gr; Tel.: +30-2810379227

S1. Raman spectra

Table S1 lists the Raman peaks of the unfilled PA12 sample which were derived from existing bibliography. By introducing ATO to PA12, the Raman lines at 1000, 1029, 1581, and 1600 cm^{-1} of pure PA12 were lost. Moreover, the broad photoluminescence across the spectral sensitivity range increased with increasing concentration of ATO.

Table S1. Major Raman peaks of PA12 pure identified and their related assignments

Wavenumber (cm^{-1})	Raman peak assignment
1000	C-H in-plane bending [1]
1029	C–C and C-O vibration [2]
1062	C-O-C stretching [1]
1106	C-O-C stretching [1]
1153	Skeletal deformation [2]
1193	C-O-C stretch [3]
1294	C-O-C stretching [1]
1435	$C-H_2$ deformation [1,4]
1581	unidentified
1600	Skeletal vibration of the C=C aromatic ring [5,6]
1632	C=C vibration [7]
2849	$C-H_2$ symmetric stretching [2]
2883	$C-H_2$ symmetric stretching [2] [8]
2899	C-H stretching [1]
2922	$C-H_2$ asymmetric stretching [2]

References

1. Stuart, B.H. Temperature Studies of Polycarbonate Using Fourier Transform Raman Spectroscopy. *Polymer Bulletin* **1996**, *36*, 341–346, doi:10.1007/BF00319235.
2. Makarem, M.; Lee, C.M.; Kafle, K.; Huang, S.; Chae, I.; Yang, H.; Kubicki, J.D.; Kim, S.H. Probing Cellulose Structures with Vibrational Spectroscopy. *Cellulose* **2019**, *26*, 35–79, doi:10.1007/s10570-018-2199-z.
3. Resta, V.; Quarta, G.; Lomascolo, M.; Maruccio, L.; Calcagnile, L. Raman and Photoluminescence Spectroscopy of Polycarbonate Matrices Irradiated with Different Energy 28Si+ Ions. *Vacuum* **2015**, *116*, 82–89, doi:10.1016/j.vacuum.2015.03.005.
4. Zimmerer, C.; Matulaitiene, I.; Niaura, G.; Reuter, U.; Janke, A.; Boldt, R.; Sablinskas, V.; Steiner, G. Nondestructive Characterization of the Polycarbonate - Octadecylamine Interface by Surface Enhanced Raman Spectroscopy. *Polym Test* **2019**, *73*, 152–158, doi:10.1016/j.polymertesting.2018.11.023.
5. Luiz, B.K.M.; Amboni, R.D.M.C.; Prates, L.H.M.; Roberto Bertolino, J.; Pires, A.T.N. Influence of Drinks on Resin Composite: Evaluation of Degree of Cure and Color Change Parameters. *Polym Test* **2007**, *26*, 438–444, doi:10.1016/j.polymertesting.2006.12.005.
6. Gatin, E.; Iordache, S.-M.; Matei, E.; Luculescu, C.-R.; Iordache, A.-M.; Grigorescu, C.; Ilici, R. Raman Spectroscopy as Spectral Tool for Assessing the Degree of Conversion after Curing of Two Resin-Based Materials Used in Restorative Dentistry. *Diagnostics* **2022**, *12*, 1993, doi:10.3390/diagnostics12081993.
7. Peris-Díaz, M.D.; Łydżba-Kopczyńska, B.; Sentandreu, E. Raman Spectroscopy Coupled to Chemometrics to Discriminate Provenance and Geological Age of Amber. *Journal of Raman Spectroscopy* **2018**, *49*, 842–851, doi:10.1002/jrs.5357.
8. Liu, X.; Zou, Y.; Li, W.; Cao, G.; Chen, W. Kinetics of Thermo-Oxidative and Thermal Degradation of Poly(d,l-Lactide) (PDLLA) at Processing Temperature. *Polym Degrad Stab* **2006**, *91*, 3259–3265, doi:10.1016/j.polymdegradstab.2006.07.004.

Disclaimer/Publisher's Note: The statements, opinions and data contained in all publications are solely those of the individual author(s) and contributor(s) and not of MDPI and/or the editor(s). MDPI and/or the editor(s) disclaim responsibility for any injury to people or property resulting from any ideas, methods, instructions or products referred to in the content.
